# *“I had no choice but to escape”*: exploring women’s trajectories from early life trauma through homelessness in Addis Ababa, Ethiopia

**DOI:** 10.3389/fsoc.2026.1762686

**Published:** 2026-07-02

**Authors:** Kalkidan Yohannes, Hannah Bradby, Sibylle Herzig van Wees, Yemane Berhane, Ulrika Persson-Fischier, Mats Målqvist

**Affiliations:** 1 Department of Women’s and Children’s Health, Centre for Health and Sustainability, Uppsala University, Uppsala, Sweden; 2 WOMHER—Women’s Mental Health during the Reproductive Lifespan, Interdisciplinary Research Centre, Uppsala University, Uppsala, Sweden; 3 Department of Psychiatry, Dilla University, Dilla, Ethiopia; 4 Department of Sociology, Uppsala University, Uppsala, Sweden; 5 Department of Global Public Health, Karolinska Institute, Stockholm, Sweden; 6 Addis Continental Institute of Public Health, Addis Ababa, Ethiopia

**Keywords:** abuse, adverse childhood experiences, community reintegration, Ethiopia, gender-based violence, pathways, trauma, women’s homelessness

## Abstract

**Background:**

Women’s pathways into homelessness are gendered, shaped by family dysfunction, economic precarity, and high rates of violence. Despite the profound implications for women and their children, evidence from Ethiopia remains scarce. This study explored women’s early-life experiences, trajectories into and through homelessness in Addis Ababa, and the challenges and resources related to community reintegration.

**Methods:**

We conducted an exploratory qualitative study in Addis Ababa, Ethiopia, in December 2023 using semi-structured interviews. Women of reproductive age experiencing homelessness were purposively recruited through a local civil society organisation (*n* = 19). Data were analysed using reflexive thematic analysis, and the report was prepared in accordance with the Consolidated Criteria for Reporting Qualitative Research (COREQ).

**Results:**

We developed four themes: “Childhood trauma from abuse”, “Sexual violence”, “Barriers to leaving street life”, and “Sources of hope”. The analysis identified three distinct trajectories – Chaotic, Circuious, and Linear – which differentiated women’s movement into and through homelessness. Women’s accounts revealed trajectories in which early harm within families and households extended into street-based victimisation. This reinforced heightened insecurity and limited their options to exit homelessness. Across themes, cumulative violence was sustained by social and structural constraints, including substance use and limited access to formal protection and justice. Despite these constraints, women emphasised sources of hope, including faith, perseverance, and aspirations to care for and support their children.

**Conclusion:**

Women’s trajectories into and through homelessness in Addis Ababa were shaped by cumulative, gendered violence across the life course. These results suggest that reintegration pathways must be tailored to specific trajectories, providing trauma-informed care for chaotic routes and economic safety nets for circuitous ones. Such an approach highlights the need for locally grounded, trauma-informed, and gender-responsive interventions.

## 1 Introduction

Homelessness among women is a complex, multifaceted issue that has gained increasing global attention (Phipps et al., 2019). While estimates vary, women are often identified as the fastest-growing segment of homeless populations (Johnson et al., 2018; Bretherton, 2020). Research consistently demonstrates that women’s pathways into and through homelessness are gendered, shaped by a combination of individual, interpersonal, social, and structural factors (Baptista, 2010; Evans and Forsyth, 2004).

These factors include family conflict and dysfunction (Phipps et al., 2019), women’s subordinate positions within patriarchal households (Baptista, 2010), social exclusion (Mayock and Bretherton, 2016; Gültekin et al., 2014), physical and sexual abuse (Phipps et al., 2019), the criminal activity of others (Evans and Forsyth, 2004), economic precariousness (Haile et al., 2020a; Nathan and Fratkin, 2018), child marriage (Haile et al., 2020a), intimate partner violence (IPV) (Johnson et al., 2018; Gültekin et al., 2014), and the responsibilities of motherhood (Phipps et al., 2019; Mayock et al., 2015). Crucially, these influences do not operate in isolation; they interact to create and sustain homelessness. Researchers therefore call for investigations that examine their interconnected nature rather than treating them individually (Bullock et al., 2020; Hess et al., 2025).

Among the most powerful—yet often underexplored—interconnected drivers are the experience of trauma in early life (Phipps et al., 2019). Adverse childhood experiences (ACEs), which encompass abuse, neglect, and household dysfunction, are disproportionately prevalent among people experiencing homelessness (Sundin and Baguley, 2015). A meta-analysis suggests that nearly 90% of homeless adults in the USA, Canada, and the UK report one or more ACEs (Liu et al., 2021). Among homeless women specifically, a systematic review found that both the prevalence and severity of ACEs are substantially higher than in the general population (Phipps et al., 2019). In sub-Saharan Africa, the situation is similarly concerning. A multi-country analysis of Violence Against Children and Youth Surveys (VACS) found that approximately 72% of females had experienced at least one form of ACE, with nearly a quarter reporting three or more ACEs (Amene et al., 2024).

In Ethiopia, national data indicate elevated rates of physical or sexual violence among females aged 15–49 (Amene et al., 2024; UNICEF, 2024), as well as high levels of gender-based violence (Mulatu et al., 2024) and child marriage (UNICEF, 2024). Beyond these national statistics, the consequences of ACEs for homeless women are profound and long-lasting. Exposure to multiple forms of abuse – physical, sexual, and emotional – is a significant predictor of complex trauma (Phipps et al., 2019) and chronic or episodic homelessness and is associated with adverse mental and physical health outcomes (Zabkiewicz et al., 2014; DeLisi et al., 2019). Poorly managed trauma is, in turn, associated with chronic homelessness and poor health outcomes (Montgomery et al., 2017), and has been linked to substance use disorders, mood and anxiety disorders, and suicide risk (Phipps et al., 2019; Douglas et al., 2010; Vázquez and Panadero, 2019).

These early traumatic experiences do not occur in isolation; they set in motion a cascade of barriers that make exiting homelessness particularly difficult for women. Research from multiple settings has identified factors that impede women’s exit from homelessness. These include having dependent children (Cobb-Clark et al., 2016), difficulties with transportation and childcare access (Thompson et al., 2020), a lack of affordable housing (Haile et al., 2020a), and the shame and stigma associated with returning to family (Haile et al., 2020a). Conversely, studies have documented sources of resilience that enable women to cope with, and sometimes exit, homelessness. These include social support (Bonugli et al., 2013), a sense of hope and positive future orientation (Moorkath et al., 2022), connection to services that foster safety and stability (Andermann et al., 2021), and personal strengths such as self-determination and religious faith (Phipps et al., 2019; Kerley et al., 2011; Phipps et al., 2021).

While these barriers are well-documented in Western contexts, their specific manifestations in low-income settings such as Ethiopia remain critically under-researched. In Ethiopia, violence against women is a major public health concern, with a meta-analysis reporting a pooled prevalence of nearly 47% for intimate partner violence (Kassa and Abajobir, 2020). Child labour also remains widespread. Globally, an estimated 160 million children are involved in child labour, according to the International Labour Organization (ILO) and the United Nations Children’s Fund (UNICEF) (International Labour Office and United Nations Children’s Fund, 2021).

Although Ethiopia has ratified international conventions and enacted national legislation, such as the National Social Protection Strategy (Ministry of Labour and Social Affairs, 2016), the Revised Family Code (FDRE, 2000), the Labour Law (FDRE, 2019), and the Criminal Code (FDRE, 2005)—enforcement of these protections remains weak. Child labour, abuse, and violence persist with impunity, and reporting mechanisms for sexual assault are often ineffective, leaving women without legal recourse.

Despite the severity of these intersecting issues, research on women experiencing homelessness in Ethiopia remains scarce. The limited existing literature suggests that childhood abuse, family conflict, poverty, and child marriage are significant pathways into homelessness (Haile et al., 2020a; Nathan and Fratkin, 2018), but no study has traced trajectories from early-life trauma through homelessness or examined barriers to community reintegration in the Ethiopian urban context.

To address this gap, we conducted an exploratory qualitative study guided by three research questions:

(1) What early‑life experiences and adverse childhood events precede homelessness among women in Addis Ababa?

(2) What are women’s trajectories through homelessness, including the role of ongoing violence and abuse?

(3) What barriers to community reintegration, and what sources of resilience, do women perceive?

To structure our investigation of these multi-level factors, we applied Bronfenbrenner’s social-ecological framework as a theoretical lens (Bronfenbrenner, 1979). This framework—elaborated further in the Discussion—organises our findings by showing how abuse functions as a “common thread” across individual, relational, community, and societal levels of influence.

## 2 Methods

### 2.1 Study design and site

We conducted an exploratory qualitative study in Addis Ababa, Ethiopia. This design was chosen to enable an in‑depth, flexible exploration of lived experiences, which is appropriate given the limited prior research in this context (Stebbins, 2001; Elliott and Timulak, 2005). Participants were recruited from a local civil society organisation in Addis Ababa, which was established through government collaboration to help mothers and their children experiencing street homelessness. The organisation offers financial support following a three-month programme that includes life skills and financial management training.

### 2.2 Study period and population

The study was conducted in December 2023. The study population consisted of beneficiaries of a local civil society organisation. Participant recruitment was facilitated by social workers and a programme coordinator from the organisation. The study population included women of reproductive age who were willing to be interviewed and could speak Amharic. Duration of homelessness was not a requirement for inclusion; newly homeless (less than a year), episodically homeless (homeless at least three times a year), as well as chronically homeless (homeless for a year or more) were included in the study.

### 2.3 Sample size and sampling technique

We recruited participants in collaboration with social workers and a programme coordinator, using purposive sampling based on the inclusion criteria. Data collection continued until thematic saturation was achieved, meaning no new insights emerged from subsequent interviews. The final sample of 19 participants provided sufficient information to address the research questions, given the study’s specific focus, the rich dialogue obtained, and the depth of case-oriented analysis.

### 2.4 Data collection procedures

The primary investigator (KY) conducted all interviews in Amharic, the official language of Ethiopia, in the organisation’s psychology consultation room. KY, a PhD candidate at Uppsala University with a background in integrated clinical and community mental health (ICCMH), had previous experience conducting qualitative interviews and studies with disadvantaged populations. Prior to fieldwork, KY completed additional training in qualitative methods to further strengthen methodological rigour. KY conducted all 19 interviews and maintained reflective field notes. No prior connection existed between the interviewer and the interviewees. Interviews covering childhood histories, drivers of homelessness, and women’s pathways through homelessness were conducted using a semi‑structured interview guide. Interview duration ranged from 22 to 60 minutes.

### 2.5 Data analysis

Interviews were transcribed verbatim in Amharic by professional translators. An independent translator back‑translated 20% of the transcripts for accuracy and conceptual equivalence. Any discrepancies were reviewed and resolved by the research team before the formal analysis. Data were analysed using Braun and Clarke’s reflexive thematic analysis (Virginia and Clarke, 2021; Braun and Clarke, 2006) following six recursive phases. Phase 1 (familiarisation): KY read and re‑read all 19 transcripts multiple times, noting initial impressions and potential patterns in a reflexive journal. Phase 2 (initial coding): KY generated line‑by‑line codes across the entire dataset. Phase 3 (generating initial themes): the research team (KY, MM, UPF, HB) collated codes into potential themes. Phase 4 (reviewing themes): the team checked themes against coded extracts and the full dataset. Phase 5 (defining and naming themes): the team refined, defined, and named the four final themes. Phase 6 (writing the report): KY drafted the results with illustrative quotes, after which all authors reviewed and approved the final manuscript.

Discrepancies in interpretation were resolved through dialogue and consensus, and KY maintained a reflexive journal throughout the analytic process. The report was prepared in accordance with the COREQ guidelines (Tong et al., 2007).

## 3 Results

### 3.1 Socio-demographic characteristics of the participants

A total of 19 participants (aged 18–49) took part in the study. Eleven women (57.9%) were between 25 and 29 years old. Six participants (31.6%) had one child, while 11 (57.9%) had three or more children. In terms of marital status, the majority were separated (*n* = 12, 63.2%), with others being single (*n* = 2, 10.5%), married (*n* = 2, 10.5%), divorced (*n* = 2, 10.5%), or widowed (*n* = 1, 5.3%). Ten out of 19 (52.6%) could not read or write, eight (42.1%) had attended primary school, and only one (5.3%) had attended secondary school. The primary source of daily income for most respondents (*n* = 16, 84.2%) was panhandling or begging. Regarding occupation, 16 participants (84.2%) self-identified as jobless, while three (15.8%) were engaged in daily labour (*n* = 2) or worked as a janitor (*n* = 1). Additionally, a considerable proportion (*n* = 13, 68.4%) were from families of more than five people. Participants reported their duration of homelessness as ranging from episodic periods of three months to chronic homelessness lasting over a decade (Table 1).

**Table 1:** Socio-demographic characteristics of women experiencing homelessness in Addis Ababa, Ethiopia (*n* = 19).

Variable	Category	Frequency	%
Age	18–24	3	15.8
	25–29	11	57.9
	30–34	3	15.8
	35 and above	2	10.5
Marital status	Single	2	10.5
	Married	2	10.5
	Widowed	1	5.3
	Separated	12	63.2
	Divorced	2	10.5
Religion	Ethiopian Orthodox Christian	10	52.6
	Muslim	3	15.8
	Protestant	6	31.6
Number of children	One child	6	31.6
	Two children	2	10.5
	Three and above	11	57.9
Educational status	Unable to read and write	10	52.6
	Primary school	8	42.1
	Secondary school	1	5.3
Occupation	Jobless	16	84.2
	Daily labourer	2	10.5
	Janitor	1	5.3
Source of income	Daily labourer and selling items on the streets	3	15.8
	Panhandling	16	84.2
Pregnancy status	Pregnant	1	5.3
	Not pregnant	18	94.7
Parents’ status	Both alive	8	42.1
	Mother alive	3	15.8
	Father alive	2	10.5
	Both passed away	4	21.1
	Do not know about the family	2	10.5
Family size of the participant	2–4	2	10.5
	Five and above	13	68.4
	I do not know	4	21.1
Duration of homelessness	10 years and above	8	42.1
	Five years and above	4	21.1
	One year to five years	4	21.1
	Less than a year	3	15.8

### 3.2 First-time homelessness

**Figure 1:**
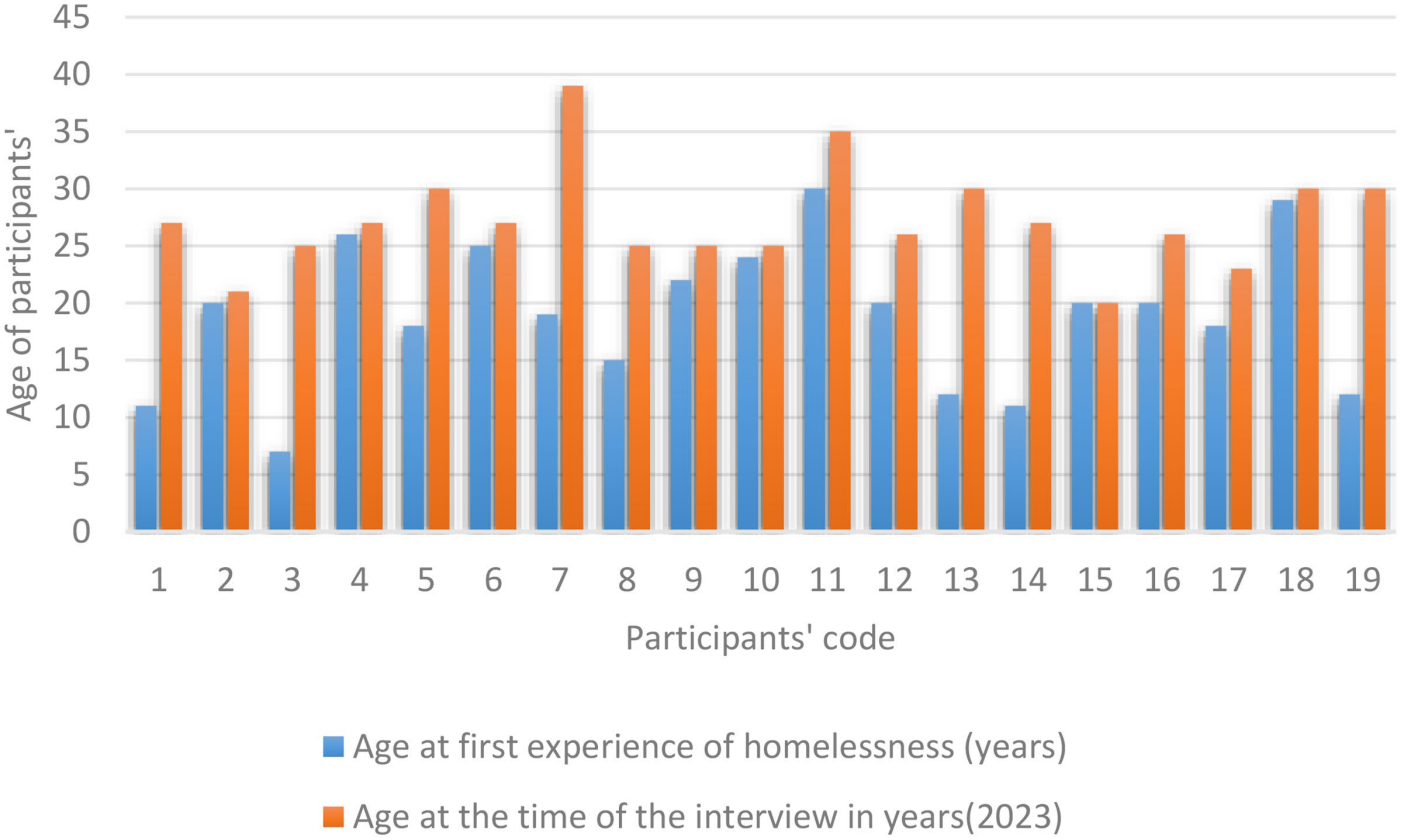
Age at first homelessness episode (blue bars) and current age at interview (orange bars) among women experiencing homelessness in Addis Ababa, Ethiopia, 2023 (*n* = 19).

The bar chart below displays the distribution of participants’ ages at first episode of homelessness compared with their ages at the time of data collection (Addis Ababa, Ethiopia, 2023). Ages are given in years. Overall, childhood and adolescence were the most common periods for first‑time homelessness. Participants aged 20 years showed a moderate frequency, whereas first‑time homelessness after age 25 was less frequent (see Figure 1).

### 3.3 Women’s trajectories through homelessness

The 19 life histories followed three distinct patterns, which are operationally defined and mapped to individual participants in Supplementary Table 1. The first group followed a Chaotic trajectory, characterised by chronic displacement and recurring loops of addiction and victimisation (e.g., P1, P3, P7, P13, P14, and P19). The second group followed a Circuitous trajectory, involving episodic movement between marginal housing and the street, often triggered by adult shocks such as partner abandonment or inability to pay rent (e.g., P2, P4, P10, P15, and P18). Finally, a Linear trajectory was observed in cases of direct transition from acute domestic crisis to the street (e.g., P5, P6). Overall, traumatic early childhood experiences, disrupted family relationships, family dissolution, and loss of a parent were common among women following chaotic pathways.

### 3.4 Themes and subthemes

**Figure 2:**
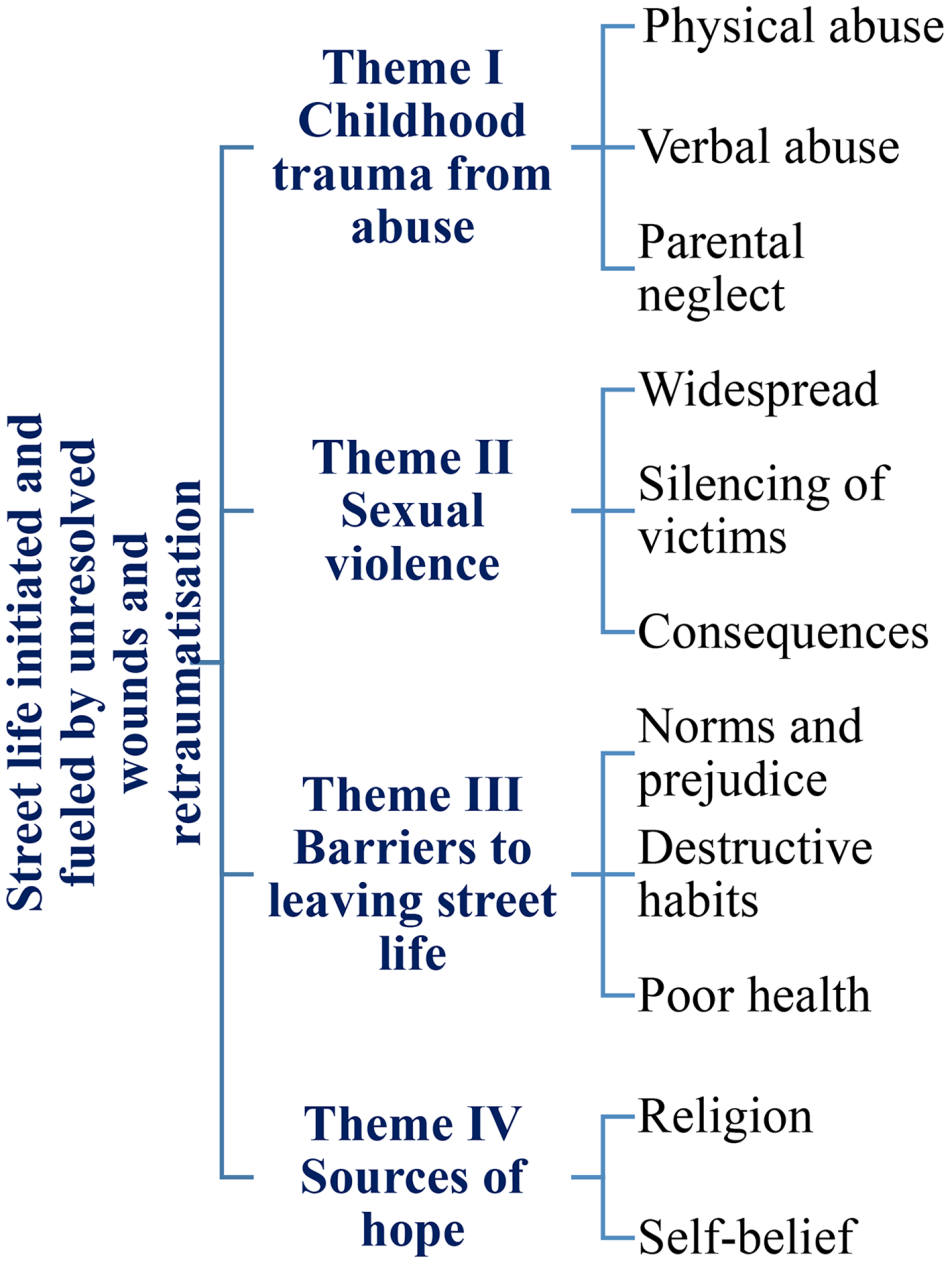
Themes and subthemes identified through reflexive thematic analysis.

We developed four interwoven themes and eleven subthemes regarding the factors influencing the drivers and trajectory of homelessness from early childhood age. Abuse was the common thread in their journeys into and through homelessness. Theme I addresses physical, parental, and verbal abuse that contributes to homelessness; Theme II focuses on abuse during homelessness; Theme III describes barriers to leaving street life; and Theme IV identifies sources of hope. Several participants attributed their first episode of homelessness to adverse childhood experiences that subjected them to physical, verbal, and emotional abuse during childhood. Early-life physical abuse, neglect, and verbal wounds (Theme I)—whether unresolved or unaddressed—were compounded by re-traumatising sexual abuse (Theme II). Multiple factors shaped these women’s plans for, and actions towards, societal reintegration (Themes III and IV) (Figure 2).

Although we focused on convergent patterns across participants’ accounts, we also noted areas of meaningful variation. Homelessness duration and episodicity differed significantly between women whose homelessness was rooted in early childhood trauma and those whose homelessness was largely caused by partner abandonment. The use of substances was not universal, some women avoided it for religious or personal reasons.

#### 3.4.1 Theme I: childhood trauma from abuse

Participants described being forced into in domestic work and multiple forms and mistreatment from their early years, which caused them to flee their family home and hometown for the streets. The first two subthemes are “physical abuse” and “verbal abuse”. In addition to physical and verbal abuse from stepfathers, relatives, and domestic employers, participants highlighted dysfunctional family dynamics, parental death, parental neglect of a child’s needs, and maternal favouritism towards other siblings, while abuse was concealed; these factors contributed to first-time homelessness (Subtheme 1.3: Parental neglect).

##### 3.4.1.1 Physical abuse

Participants who had been subjected to child labour while living with their close family or relatives described being trapped. Recruited as domestic workers in some cases, they were too young to perform the traumatic domestic work required for extended periods:

*“Then, a woman approached me and asked what was wrong with me. I explained to her I had left the house because my aunt beat me. Then I told her I want to work, even without payment, as long as she gives me clothes. I told her I am hungry, and she takes me with her. Then she asked about my story, and I explained it to her. Here, the cattle eat the sludge settled by the fermentation of traditional liquor, so I used to carry 20 litres of it. Twenty litres is very heavy for me at age seven. She mixes the sludge, and the cattle will drink it. She also used to make me pull out a pot of water from the well with a rope, and then I got ill, but she could not understand my pain. She insists that I do more without rest.” (P17)*

Discussions regarding the child labour they were forced to perform at their families’ homes or as domestic workers during their early years were filled with painful memories. As illustrated in the extract above, when the participant said, *“…she could not understand my pain…”* and *“…she insists I do more without rest…”*, she described the multiple types of child labour and cruelty she endured.

Concerns about early life experiences and the ongoing occurrence of physical and verbal abuse were common. The situation became more complicated when the biological father was the abuser. The women emphasised the violence that resulted from social and relationship problems within the family as a driving factor for their homelessness. Some participants recalled how parental alcoholism led to abusive behaviour towards them, and witnessing maternal abuse further exacerbated the situation:

*“Yes, I remember; the reason was that my father used to beat me a lot at home… He used to drink at night and beat me… because he was drunk. He used to abuse me so badly… He used to beat me a lot. He also had no peace with my mum and many people, so I just left” (P3)*

In addition, some participants stressed that harsh physical punishment was a precipitating psychosocial factor for ending up on the streets. For many of them, physical punishments that led to physical harm escalated after moving from the stepparent’s home to the uncle’s or grandparent’s place while they were pre-teens.

They escaped from the painful punishment to survive and ended up as a last resort living on the street:

*“I was not capable of handling more abuse than that, and I ran away from my home (my parents’ home in Addis Ababa). I left home; my grandfather found me and took me to Shashemene (a town in the Oromia region). When I lived in Shashemene, my grandma was abusive. She (my grandmother) often hit me. It was she who forced me to work as a slave. She frequently hit me. A neighbour always kept me hidden from her. One day, I ran from home when my tolerance was exceeded; I had no choice but to escape. It was to avoid getting hit by a stick or hand. The fear of pain and being whipped… Having my head forcibly dipped in water, being beaten, and being dipped in water when I make a mistake is quite scary. I had no other option but to flee. The fear of pain and being beaten. It is my grandmother. She punished me by using berbere (Ethiopian red pepper spice mix), and she fumigated me with berbere smoke.” (P19)*

Another participant pointed out that physical abuse by her sibling was a predisposing factor for her first episode of homelessness. She explained that her mother favoured her elder brother and allowed him to abuse her:

*“My mum did not try to stop my brother from beating me. She always sides with him, like a faction in the family. Three siblings were with my mum (Inaudible voice). If our dog could speak, he would be trusted more than me. And I was the only victim of his nastiness. We (her other siblings) are the victims of his brutality… I cry when I think that I am where I am because of my brother. If my father were alive, I would not be on the streets. I told them that and went away.” (P14)*

In this extract, the participant felt that the death of her father created an environment for her mother to favour her brother while ignoring her daughter’s suffering and giving him the freedom to beat her.

This led to her ending up on the streets at age 11, seeking freedom and escaping an unshielded, unprotected, and neglected home life (see Supplementary Table 1). Rape, life‑threatening circumstances, and chronic cycles of homelessness followed.

##### 3.4.1.2 Verbal abuse

The analysis revealed that, in women’s early lives, physical and verbal abuse from relatives and/or domestic employers were intertwined. Experiencing these coupled abuses left them feeling trapped when deciding whether to stay and endure persistent abuse while being told they were unwanted, or to leave for the previously unknown street life. They faced a choice between enduring daily verbal abuse and continuing to attend school or leaving their stepfather’s or relative’s house.

One participant who had experienced such adverse childhood events chose to leave her stepfather’s home as the lesser of two evils:

*“My stepfather is an evil man… He always returned home drunk. He used to ask, ‘Why did I see you? Why didn’t you work?’ He did not want me to play with the kids (stepsisters and stepbrothers), either. He calls me a ‘bastard’ (a child born outside of a marriage). He also beat my mother; he regularly punched her and frequently said, “I am raising your bastard.” Having been beaten daily by him, I left my exercise books at home and ran away.” (P19)*

##### 3.4.1.3 Parental neglect

The analysis revealed that four participants were not yet 10 years old when they were left to live with others because of family disintegration or because their mothers remarried (the new husband often later became the woman’s stepfather). Among them, one participant described being abused by an aunt and attributed her situation to being abandoned by her mother at an early age:

*“My father passed away when I was little; I recall nothing. And my mother left me with my aunt and married another husband… My aunt did not even send me to school because I had to do all the domestic work, and I was hurting a lot. (…) When my mother married, she did not want to take me to her new spouse’s place. I have a brother. My uncle took him to Addis Ababa, and she left me there [with my aunt]. So, I went out of the house when she hurt me so much.” (P17)*

Conversations about family histories were challenging and distressing for many. As the quote above shows, the participant states, ‘...left me with my aunt and married another husband...’ and ‘she did not want to take me with her...’. She specified how she was denied love and how the physical absence of her mother emotionally affected her.

Other women, who also witnessed marital discord and the early loss of their fathers (quite a few of them lost their fathers), or whose parents maltreated them, conveyed that they did not even know what family love was and lived with the feeling of being disliked by their parents. One participant questioned the reasons for the absence of parental care and follow-up, and she expressed her feelings of frustration about the future of her child as follows:

*“Why am I wandering in Addis Ababa while my mother and father are still alive? If they die, it is all right, but while they are alive, that is not okay. Why did they break up, and why did all this happen to me? I do not like them both. I hate them (*…*) because after I was born, why did they separate and give birth to someone else? And I thought of my daughter; she will suffer the same way I suffered in the future.” (P10)*

Another woman reflected on her family’s failure to prioritise sending her to school or showing her love, and how this negatively affected her life:

*“Because my family did not treat me well while my friends were learning, they did not send me to school; I stayed home. I was not educated as a child in my youth, and I felt bad when my friends came home from school. Even now, I have a grudge against my family. Because I cannot even write my name today; if they had taught me then, I would not be like this now. At least I could write my name. They did not teach me; they did not give me love.” (P7)*

Some participants voiced early experiences of not being seen or cared for by their parents compared with their siblings. This feeling, combined with parents’ favouritism, shaped their belief that they did not belong in the family home but rather on the streets, where they could find freedom.

#### 3.4.2 Theme II: sexual violence

The second theme, *“Sexual violence”*, included three subthemes: *widespread, silencing of victims, and consequences*. Many women spoke about their traumatic experiences and the damaging aftermath of sexual violence. Some discussed the social and cultural norms that prevented reporting sexual assault (rape), leaving them to suffer its effects.

##### 3.4.2.1 Widespread

Data analysis revealed that 17 out of 19 participants (89.5%) had experienced various forms of sexual violence, including sexual harassment, sexual assault, rape, sexual exploitation, and child sexual abuse. Discussions of past and present sexual violence were prominent, particularly experiences of gang rape:

*“Then I started spending nights in Giorgis (a neighbourhood in the city). In the middle of the night, I was raped and gave birth to that lost child. I don’t know who raped me; I was raped by six men (mumbling). They took me and raped me, then I gave birth to my child.” (P3)*

In addition to rape, child sexual abuse was a prominent issue among several participants. One woman described how and when she experienced rape in her early years and expressed relief that she had escaped gang rape:

*“I was 13 years old. A man raped me. It was painful and traumatic. Back then, I did not know about men. During that time, it was dark, and I was very friendly to them (street dwellers); however, he forced me into a makeshift shelter and raped me. The pain followed. I was a little girl. At that time, I had no understanding of what menstruation was. After being raped, I bled profusely. He was older than I was. He (the rapist) was around 30 years of age…. Thank God… I was only raped by one person. There was only one person who raped me… I just came in bleeding at the churchyard.” (P19)*

Sexual crimes committed against participants, many of whom were mothers, were among the most common stressful life events on the street. One participant reflected on how she allowed herself to be raped because resisting or struggling would be distressing and unsafe for her children. Her children slept beside her while she endured the pain of rape to ensure their safety:

*“It happened at night; I slept with my children, and he (a rapist) came and grabbed me while I was sleeping. That was the reason I have now conceived a baby. When he came and grabbed me, I just let him rape me at that time so that the children would not suffer from what they may see. I just let him rape me.” (P4)*

A chronically homeless woman (for 20 years) shared her experience of how her female friend betrayed her and orchestrated her rape, which caused her unwanted pregnancy and subsequent domestic abuse during pregnancy:

*“Before that, I went through a rape, and it was a woman who approached me, saying she would give me a place to stay, and she gave me away to two boys.” (P7)*

Discussions regarding the extent to which harassment was spread, how women normalised it and got used to it, and how they expected it to happen daily were described by a participant who had been living on the streets since the age of 11 as follows:

*“I can’t sleep. We don’t sleep like a human. If you sleep, the men will need you for something else. We cut our hair short and wear hats or scarves every day. If they see us lying down and know we’re women, they’ll rape us. Everyone, even drunks. We live by looking like men. I mean… We’ll eat anything we find. We’ll just fall asleep if we can’t.” (P19)*

Contrary to these statements, some women felt that they were not as vulnerable as other rape survivors because of their living area, where *“fearless women in the area”* also lived: homeless women whom men feared.

##### 3.4.2.2 Silencing of victims

Many women spoke about their sexual assault experiences, the aftermath and complications of rape, and their perceptions of the taboos and outcomes of disclosing their experiences of sexual assault and suffering:

*“It was taboo to talk about rape. I could not tell anyone. As a result, I had to remain quiet. I did not inform anyone. I did not say anything. It took me a long time to overcome my pain and move past it [cried silently].” (P19)*

Most participants did not feel they could ask for help after rape and other forms of sexual assault, primarily due to their family’s silence about it, the normalisation of the practice (as they learned after unsuccessful attempts to file a sexual assault case with the police), and fear of the case being heard by their family.

*“There were two girls with me. When we woke up in the morning, we were naked, and there were men with us. Then we reported it to the police, and they were arrested. When the police officers asked them, they denied the accusation of rape. They did not admit their deed. No one could give us a solution. Also, the police did not give us a solution. One day, they would say that the suspects are not in custody. Another day, they would say that they were advising them. Then, I stopped following the case because I feared my family might hear about it. So, they will do something to me if they hear about the case. I fled from that neighbourhood.” (P14)*

Being raped by a relative or a neighbour forced a few participants (three women) to remain silent about their suffering and let the abuser go free. The perpetrators sexually assaulted female children and lived peacefully without being punished for their crimes. Meanwhile, survivors were forced into pregnancy by relatives and to raise children in hardship, living in fear and shame due to threats from their abusers.

##### 3.4.2.3 Consequences

Unwanted pregnancy resulting from rape was a common phenomenon among participants. Most women revealed that they were subjected to a multifaceted burden of sexual violence. One participant shared her street-life experience of witnessing sexual violence, specifically rape against pregnant women, which resulted in their death:

*“There are those who have been raped while pregnant. Many women died because of rape, and some women were raped while pregnant and died because of it.” (P17)*

Many women spoke about stressful experiences, the consequences of unwanted pregnancy, and adverse experiences during pregnancy, childbirth, and the postnatal period:

*“I did not know that I was nine months pregnant; the day she (her neighbour) said she would take me tomorrow, while I was sitting at home, I felt labour pain. In the morning, the lady went to church and told me to sit at home and wait for her until she came back. It was the day St Michael’s holiday was celebrated. She closed the door on me and went to church. However, I gave birth in her house until she came back…… I gave birth by myself—alone until she came from church. It took at least an hour from the time I gave birth until the lady came. Meanwhile, my baby and placenta were on the ground, and I was bleeding too.” (P17)*

Another woman reflected on how her rape incident caused unwanted pregnancy and subsequent domestic abuse during pregnancy:

*“They raped me, and as a result, I became pregnant with my first child. Then I was sick, and I was cured, and I entered another woman’s house for free. Why? Due to a housing issue, I was granted free entry. While I was working for free, the woman would not feed me while I was starving and tormented. I used to live in the kitchen, and when I was hungry, I jumped out of it over the fence and went out to the street again.” (P7)*

There was discussion about past and present sexual violence, especially repeated rape, which caused much suffering and led to suicidal behaviour:

*“It (being raped) was disgusting. I had lost interest in life. I tried to be hit by a running car. But my friends convinced me not to commit suicide. They said they were raped too, but they were living in the hope of a better life in the future, and it was okay. After I thought through what they had said to me, I stopped thinking about committing suicide.” (P14)*

There were concerns regarding how partners deceived women and how women were willing to reconcile after men returned suddenly, even when those men were the reasons some women became homeless. For many, this contributed to episodic and chronic homelessness, alongside an erosion of trust in men. Although abandonment during pregnancy caused suffering, some women perceived being with a man as protection from street life, so trying to live together despite past sacrifices was seen as part of life. In some cases, intimate partner violence and being forced to undergo intentional abortion left women feeling used, discarded, and vulnerable.

#### 3.4.3 Theme III: barriers to leaving street life

The third theme, *“Barriers to leaving street life”*, included three subthemes: norms and prejudice, destructive habits, and poor health. Participants described individual factors (substance use, health problems, and negative feelings about family and personal weakness), as well as social and cultural factors, as hindrances to integration.

##### 3.4.3.1 Norms and prejudice

Many women perceived returning home after being abandoned by their partners or losing a loved one, and, of course, having children without a formal marriage, as a shameful, socially awkward, and disgusting decision to make. They believed they would be considered to be looking for a family inheritance.

*“He (my father) was fine. He is dead now; he is not alive. I have a stepmother. They will not let me have a relationship with them because they think I will share or take away their portion (property); I do not go to them either. In our culture, returning to your family after having children is considered shameful. It is difficult.” (P14)*

Two women claimed that returning penniless to their family was difficult, grounded in the belief that no one wants a person with no money, especially if they have children to care for.

*“No, I did not. I cannot afford the transport fee. You would like to go to your family when you have something. You do not want to go there being poor. Even your family would accept you when you have something but will not accept you when you are poor. Besides, the most challenging thing is that I now have children.” (P8)*

*“Now, after all this time, yes, I want to see my father, sisters, and brothers, but when you go, it is hard to go empty-handed after all this time. It is difficult to meet the family empty-handed. I am the will of God. I want to work, and I will be glad to visit my father. I will be happy to see my brothers and sisters. I do not want to move away from them. They are my family, after all. Now, I have learned: family is important. I still hope to visit them.” (P4)*

For many participants, accessing work was hindered by their gender. The extract below shows how society’s misconceptions about women street dwellers affect their access to jobs:

*“They (the community) say it is okay for men to be “hoodlums” (ዱርዬ—in Amharic). They don’t know what we are suffering from. They call you a hoodlum because they think you’re wild. They prefer to let men work for them. They provide it to them. And they let them work. However, no one wants the women street dwellers to work.” (P19)*

Furthermore, some shared their experiences of witnessing social integration problems that were personal as well as economic and cultural challenges associated with childhood neglect:

*“All my family doesn’t want me to take my children with me. They are wealthy. They do not even like someone poor. For that reason, I am not eager to see my family. I prefer someone who is not my relative. You know why I say so? They did not show me a good life.” (P5)*

While some participants had no place to return to, others were from wealthy families and were searched for and taken back by their families. However, some were reluctant to break the cycles of homelessness, and some could not see a way out due to mental health issues, as one participant noted:

*“Living on the street is an ugly thing. By the way, some people start living on the street without any good reason. Some people start living on the street because of difficulty. Some have a rich family and live on the street. Their family will come and take them, but they will come back. Did you get what I mean? Some are educated and live on the street because of depression.” (P1)*

##### 3.4.3.2 Destructive habits

Substance use was reported by a subset of participants, while others never used substances (because of their religion, a belief that a woman should not use drugs, to keep the money to raise their child). P8 stated:

*“I do not have this kind of thing from the beginning, and I do not have any addiction. My desire is only to find food so that my children do not starve, but I have never used drugs”.*

In contrast, other participants described initiating substance use as a coping mechanism for street life. A participant stated that A participant described how she and her friends initiated and persisted with substance use even after completing a rehabilitation programme to manage “street life”, especially in harsh situations such as cold winter nights, sexual harassment, and socialisation with other people who live in the area:

*“I started it (substance use) as a joke. So, when they smoke, there is something [glue] for the cold weather. Did you get what I mean? I started chewing khat and a cigarette together. After some time, I stopped using it during rehabilitation. But I did not stop once and for all. I could do it once a week or twice a week. Do you know how I started drinking alcoholic drinks? Since we spent the whole night walking around…” (P1)*

Many participants felt that coping with “street life,” particularly during harsh situations like cold winters, or to escaping sexual harassment and socialise with locals led them to initiate and persist with substance use.

*“And she (my friend) smokes and chews khat, then goes out to her business, and I love her so much, but she taught me how to do things. Smoking cigarettes…. Moreover, I got used to smoking cigarettes. I tried what the khat tasted like and said, “Isn’t this bitter?” Then she told me I would get used to it, so I started doing everything she did. She (my friend) said, ‘It will protect you from the cold. Even if it rains heavily, it will not affect your body’”. (P3)*

Although 16 out of 19 participants (84.2%) underscored that they were not involved in survival sex and considered it a taboo (a few of them had multiple sexual partners, though), one participant described how some women survive on the streets and how she was involved in it:

*“Then, I started a business [sex work] and I later understood that business means spending the night with a man… and a man comes with money, gives me money, then [inaudible] and then takes my money… We stand there until midnight; even with heavy rain, we will be standing” (P3)*

In contrast, another participant shared her opposing view regarding involvement in survival sex, but she did not connect it to taboo but to a personal stance:

*“Some say that we should work like them. I swear they even say that they’re earning more. There was a friend of mine; she is a prostitute now. Her plastic shelter is located in Doro Manekiya, Arada (a neighbourhood name). My friend told me about that, but I said I was uninterested. She lives like that. She earns by doing that. She had one child, so she covered his expenses. But I said that I didn’t want it. I prefer to suffer till the day God has destined.” (P12)*

##### 3.4.3.3 Poor health

Some participants expressed their experiences of being vulnerable to physical health conditions as well as reproductive health concerns. Commonly mentioned medical conditions included sexually transmitted infections, typhoid fever, severe back pain, kidney issues, and the suffering of their children from neurological conditions, mental health problems, and food poisoning. One participant described how she was suffering from back pain as follows:

*“As you can see [she pointed to her back], my back is sick. It is a serious matter. They sometimes ask me to bring water to do this, but I cannot. My hip hurts so much that I cannot stand for long hours. And this all happened because of that abortion. This is how it affected my health.” (P3)*

Three participants had suicidal ideation, suicide attempts, death wishes, and thoughts of harming their infants, alongside depressive features. Two participants shared their suicidal intentions and how they felt about themselves as follows:

*“I struggle with self-hatred due to the bad luck I have faced since childhood. And I doubt that things will improve. I do not think I will be someone anymore. No one wants me. A relative ran away from me.” (P17)*

*“The reason why I hate myself is that I am like this because I have had bad luck since my childhood. And I do not think it will be fixed after that. I do not think I will be someone anymore. Nobody wants me. A relative ran away from me. I went out into the streets. I have tried everything to die. I’ve drunk poison, and it has not killed me; it just burnt me inside. I’ve thought about many things. Even when I chat with my friends, I ask them to tell me any poison that will kill me. They said mixing soap, paracetamol, and some leaves would kill me in a minute. I tried such things when life hit me hard, but it didn’t kill me.” (P18)*

Although participants had suffered from stressful situations, 18 out of 19 women (94.7%) had no experience of infanticide or aggression towards their newborn babies.

A young mother shared her mental health experiences and infanticidal behaviour after giving birth:

*“When I am sad, I just cry. Like I said earlier, it will be directed at my son when I am angry. I take it out on my son. I promise that my son will express gratitude once I leave. When I get angry, I do not act like I used to. Before, when I got angry, I would lift my son from where he was sleeping and throw him from above; he would cry and say nothing… He did not know anything at that time. I would say that he would better understand things now. He does not do anything other than cry. Then, I regret it immediately. When he cries and does something, I will cry too and try to forget all the problems. When I see him cry, I say to myself, “He is innocent, and he does not know anything,” and I regret what I have done and cry.” (P2)*

In this study, 18 out of 19 participants did not have health insurance to cover their medical expenses, making early treatment more arduous. Complexity was added when their physical problems were accompanied by their children’s nutritional deficiencies, safety concerns, and deteriorating health status (epilepsy, mental health conditions, as well as rape exposure).

#### 3.4.4 Theme IV: sources of hope

##### 3.4.4.1 Religion

Participants stated that the factor that helped them most to cope with the stress of street life was the sense of peace they felt in a religious place and praying for the creator’s support. Although some felt betrayed or abandoned by God, many reported feeling more protected by the creator than by the law or their own families.

*“What I thank God for is that I have never been addicted. I refrain from smoking, drinking, or chewing khat, and I avoid the smell of glue. In general, I am free from any kind of addiction. However, I used to pray to get out of this kind of life and have a decent one. I used to pray to God like that” (P12)*

Positive religious coping was observed among some of the participants. One participant shared her religious experiences and how they helped lift her emotions:

*“It is only God who solves all these problems. The church is a big house for all of us. Many people talk about their suffering stories being changed. It is shocking when you hear a miracle. You pass through injuries, failures, many problems, and sufferings, and there is something called God. So, I am an Orthodox Christian. I go to church. That’s it. I went there to join the community; my spirit was calmed there, and I became a woman of order. I enjoy going to church” (P6)*

##### 3.4.4.2 Self-belief

After being exposed to street life—characterised by multidimensional violence, abandonment, and the inability to fulfil basic needs for themselves and their children—the participants felt that their experiences had equipped them to use their insights to help other street-living women break the cycle of homelessness. Participants emphasised their mental readiness to leave street life and become self-sufficient to improve their own and their children’s lives:

*“We sometimes pay twenty birr every two weeks. At the time of my mother’s death, women in the neighbourhood collected some money. They gave it to me and asked me to join them in the edir (Edir is a funeral association that manages ceremonial occasions, marriages, and funerals). Then I said to myself, “They gave me money during my mother’s death; I should join them”. Since then, thank God I have been a member of that edir. On the thirtieth day, money is collected from each member so that we can use it to prepare dinner when a death occurs among us. We also collect another twenty birr every fifteen days to save because I want to work” (P3)*

Some interviewees described their strengths as a positive outlook and believing in themselves since they are important to their future selves and their children’s future:

*“It is all in Allah’s hand. I do not know what the future will bring. If you work hard, you won’t fail as a human being. If someone works hard, they will live the life they desire. They won’t crawl on the dirty streets. I will be motivated to work from now on because I don’t want my children to beg like I do”. (P8)*

*“My strength is that I am a hard worker; I used to work a lot. I used to work a lot. I work hard. Also, before I gave birth, if there were anyone in need, I would help them; I have so much pity for people. I want to change myself. I used to help my family, and I used to fulfil all my own needs”. (P9)*

Some women emphasised that accepting the situation and transforming sad circumstances into happiness was a way to improve the situation:

*“I do not let anyone take advantage of me. My children are also well-behaved. For my part, I am not like a street dweller. It’s not that I don’t smile at anyone. The most important thing is your demeanour. Living with people requires that you maintain good behaviour. All you have to do is accept everything that comes. All you have to do is be happy, and you will turn sadness into happiness. And you will cope with problems and illness unless they become severe; you will cope. You will be strong if there is no one around you. If there is someone around you, then you will get lazy. But if you are alone, you will be stronger. I do not have anything more to say. Now, when I stopped the job (washing clothes for people) due to a medical condition (a kidney disease), my children started to suffer, so when I ran out of choices, I came here after hearing about an organisation that helps people [chuckle].” (P11)*

The participant noted that despite a lack of social support, she could cope with adversities. Her prioritisation of self-determination and emphasis on peaceful relationships over idleness was likely influenced by her personality. Together, these four themes illustrate how abuse, violence, structural barriers, and individual resilience intertwine in women’s trajectories through homelessness.

## 4 Discussion

Guided by Bronfenbrenner’s social-ecological framework (Bronfenbrenner, 1979), this study aimed to provide insight into the early life experiences of women experiencing homelessness, their trajectories through homelessness, and the challenges inherent in community reintegration. The research identified four interwoven themes, with abuse forming a common thread throughout the homelessness journey.

The results demonstrate the long-term and recurrent nature of traumatic experiences reported by the participants. Women’s trajectories varied considerably: those whose homelessness began in childhood due to family abuse and neglect followed chaotic, prolonged paths often lasting over a decade, while those who became homeless in adulthood following partner abandonment experienced shorter, more circuitous routes with multiple exits and returns to street life.

In addition to experiencing abuse during their childhood and pre-teen years, most of these women were survivors of various forms of sexual violence, including harassment and rape—by family members or through gang rape—and extreme cases of repeated rape. This sexual violence did not cease upon leaving home; for most, it continued and became a feature of street life. Individual vulnerability initiated women’s homelessness, and family dynamics, as well as community- and society-level factors, shaped its origins and impeded exits.

### 4.1 Individual factors

Core individual-level drivers of participants’ homelessness were described as stemming from childhood neglect and the experience of multiple types of trauma (verbal, physical, emotional, and sexual). Witnessing maternal abuse and exposure to child labour were also root causes of homelessness among the women. Previous studies on adverse childhood experiences as drivers of homelessness have supported these findings (Phipps et al., 2019).

Women reporting early-life sexual and physical brutality (e.g., P19) described prolonged, episodic homelessness, suggesting these early experiences may entrench street life. Previous studies by Broll and Huey (2020) and Young et al. (2017) have shown how a history of abuse significantly predicts multiple episodes of homelessness and recent physical and sexual violence. At an individual level, substance use (khat, alcohol, cigarettes, and, in some cases, glue) was contributed to the continuation of street life for some of the women; it served as a coping mechanism to help them endure the hardships of the streets and socialise with other street dwellers.

However, it also exacerbated their problems, leading to a relapse into previously managed or discontinued substance use upon their return to the streets. For some of the women, using stimulant drugs helped them stay alert throughout the whole night to avoid rape, other forms of harassment, and theft. These findings are supported by studies from Ethiopia (Haile et al., 2020b), Spain (Guillén et al., 2020), Australia (Thomas and Menih, 2022), and the USA (Upshur et al., 2017), which reported substance use as both a driver of homelessness and a coping mechanism for it (Thomas and Menih, 2022), as well as demonstrating the increased prevalence of substance misuse among street populations (Guillén et al., 2020).

### 4.2 Micro-level: relational factors

The homelessness journey of participants was also shaped by relationship-linked determinants such as the loss of a family member in early childhood (especially before the age of 11), parental substance abuse, partner substance dependence, family breakdown, and a family environment characterised by parental favouritism and emotionally or physically absent parents. Previous studies have identified family dysfunction, the loss of a parent in early life, and feelings of neglect as determinants of the decision to leave home at an early age and as risk factors for major depressive disorder. The findings of this study demonstrate that economic dependence was a key factor keeping women in abusive relationships, as described by participants who endured violence to maintain shelter for themselves and their children (P4, P8).

These findings are consistent with previous global studies showing intimate partner/domestic violence as a significant predictor of women’s homelessness (Mayock et al., 2016) and familial substance abuse as a driving factor of homelessness (Rotich et al., 2012).

In this study, relationship-related factors also played a significant role in the initiation and continuation of substance misuse (khat, alcohol, cigarettes, and glue), an issue that has received widespread research attention. Despite this, some participants had no history of substance use and avoided socialising with those who did, a finding Started by a study from Brisbane, Australia (Rotich et al., 2012).

These interpersonal networks (family, carers, peers, and partners) were also a source of exposure to sexual violence, as women reported abuse by close family members, neighbours, and strangers. A common theme in participants’ accounts was the prevalence of rape on the streets. Researchers from Ethiopia (Misganaw and Worku, 2013), Democratic Republic of Congo (Falb et al., 2023), and Spain (Vázquez et al., 2025) have previously reported a high prevalence of sexual assault and domestic violence among women and its correlation with previous histories of childhood abuse.

However, there has been disagreement in the literature regarding whether living on the streets itself predicts a high prevalence of sexual abuse (Weinrich et al., 2016). For instance, Weinrich et al. (2016) reported higher percentages of sexual trauma among women living in safe homes than among women in safe parking sites. The possible reasons could include differences in setting definitions, urban area safety, and small sample sizes.

### 4.3 Macro-level: community-level and societal factors

Traditional community and societal beliefs regarding the gender roles of women, as well as the acceptable treatment of domestic workers, were often treated as socially constructed norms. Many victims of household abuse were treated inhumanely, and domestic employers were left unpunished for their actions; this was normalised. Such normalised abuse meant that victims were blamed for ending up on the streets and seen as hoodlums.

Prejudice against women experiencing homelessness was a common societal phenomenon. Instead of recognising the deep-rooted predisposing and precipitating factors for homelessness, these women were labelled as disreputable, socially deviant, or “wild” when they appeared on the streets. In addition to having been exposed to abuse in early life, participants were vulnerable to new and persistent abuse, interwoven with structural factors such as poverty and reliance on relatives, stepfathers, domestic employers, and partners for their own survival and that of their children. Neighbours’ silence about witnessed abuse also reflected a lack of intervention.

The societal factor that acted as a driver of pathways through and out of homelessness was the cultural norms that implicitly supported violence and hindered community integration based on notions of losing an “ideal/proper woman” (Bretherton, 2017; Ebila, 2015).

The stigma attached to being a single mother was a source of profound shame and a perceived barrier to social reintegration, with women expressing fear of community judgement (P14). These norm-centred beliefs restricting women’s exit from homelessness and heightening fear of prejudice have also been reported by other authors as impediments to community reintegration (Skosireva et al., 2014; Moensted and Day, 2025). Moreover, policy and regulation gaps should be highlighted in this context. Ethiopia endorses and implements national policies and guidelines such as the National Social Protection Strategy (Ministry of Labour and Social Affairs, 2016), the Family Code (FDRE, 2000), the Ethiopian Labour Law (FDRE, 2019), as well as continental (Organization of African Unity, 1990) and international legislation and conventions (International Labour Organization, 1973; International Labour Organization, 1999).

There is little de facto protection for children, and child labour remains common (Kyei-Gyamfi, 2025; Abdullah et al., 2022). Previous research from Ethiopia, other African countries, and Asia supports these findings, showing that women experiencing street homelessness had previous exposure to different forms of abuse (Nathan and Fratkin, 2018; Abdullah et al., 2022; Sohel et al., 2024). Although most participants were sexually exploited, and in some cases their children were also raped, they did not get justice for reported rape cases; many did not even file a claim, having normalised rape and other forms of violence as part of street life.

This finding aligns with studies from Ethiopia describing the normalisation of rape (Presler-Marshall et al., 2020), as well as studies from Tanzania (McCleary-Sills et al., 2016), and Los Angeles (Huey, 2016) reporting women’s acceptance of violence as a normal part of life and a failure to report it to the police. Furthermore, some participants did not know their rights; they ran away from home but did not seek legal intervention. Inadequate governmental response to child labour also failed to deter abusers. Street-life struggles, cultural stumbling blocks, addiction, social stigma, lack of social support, and poor health blocked exit even when women were directed to support for reintegration into the community.

Studies from Ethiopia (Haile et al., 2020a), South Africa (Olufemi, 2000), Australia (Phipps et al., 2021), the United States (Broll and Huey, 2020), and quantitative studies from Australia (Phipps et al., 2021) and Denmark (Christensen and Vinther, 2005) have shown how stereotypes and social exclusion act as barriers to exiting homelessness and contribute to poor mental health conditions.

As already stated, there was a glimpse of hope in women’s positive religious coping and self-reliance, which may help them eventually exit street life. These findings are supported by previous studies that reported resilience (Phipps et al., 2021), viewing homelessness as part of a journey, and continuing to hope for re-joining society (Phipps et al., 2019).

### 4.4 Limitations of the study

We acknowledge several limitations. First, we recruited from a single civil society organisation, which may limit the transferability of our findings to women experiencing homelessness who are not connected to support services. Future research should consider diversifying recruitment sources, such as including street-outreach services, shelters, and drop-in centres, to capture a broad range of experiences. Second, we excluded women who did not speak Amharic and those without children; we may therefore have overlooked certain participants and overlooked important aspects of women’s street life experiences.

Finally, our thematic analysis emphasised shared patterns across participants. While this was a deliberate analytic choice aligned with our research aims, it may have underplayed variation in trajectories. Future studies could usefully employ case-based or typological approaches to explore divergent experiences in greater depth. It should be noted, however, that considerable effort was made to capture lived experiences, including those of newly homeless women (homeless for a minimum of three months), episodically homeless women, and chronically homeless women (homeless for up to 20 years), women with dependent children, those who were pregnant or in the postnatal period, women who used substances, and those with comorbid medical conditions.

## 5 Conclusions, policy, and practical implications

This study, discussed through a social-ecological lens, demonstrates that pathways into and through homelessness among women in Addis Ababa are shaped by abuse as a common thread (Themes I and II), reinforced by multi-level barriers (Theme III), and tempered by limited but crucial sources of hope (Theme IV). Women’s trajectories varied: those with early childhood trauma experienced chaotic, prolonged homelessness, while those whose homelessness was precipitated by partner abandonment in adulthood followed shorter, more circuitous routes. The findings underscore several evidence-based implications:

1. Individual/relational level: The pervasive history of ACEs (Theme I) and ongoing sexual violence (Theme II) — where rape was normalised and justice elusive — underscores an urgent need for trauma-informed mental health services coupled with accessible legal aid and violence-prevention programmes that are accessible to and tailored for women in street-based settings.
2. Community/societal level: Stigma and gendered norms that frame women experiencing homelessness as “hoodlums” and make family return a source of shame must be countered through targeted community awareness campaigns. Such campaigns should be integrated with gender-responsive social protections that address economic precarity.
3. Policy level: Gaps in justice, evidenced by unpunished child labour and ineffective police responses to rape, point not only to weak enforcement but to a systemic failure to protect the most vulnerable. Policy must bridge the chasm between de jure protections and de facto impunity, which requires mandatory training for law enforcement and a victim-centred overhaul of reporting mechanisms.

In summary, addressing women’s homelessness in this context requires a coordinated, multi-level strategy that prioritises trauma recovery, economic opportunity, and systemic protection from violence and chronic homelessness.

## Data Availability

The original contributions presented in the study are included in the article/Supplementary material, further inquiries can be directed to the corresponding author.
