# Supplementary material for: “I had no choice but to escape”: exploring women’s trajectories from early life trauma through homelessness in Addis Ababa, Ethiopia
Source: Front Sociol. 2026 Jul 2;11:1762686. doi: 10.3389/fsoc.2026.1762686 (PMC13383843; doi:10.3389/fsoc.2026.1762686)
Supplement: Supplementary file 1 [file Table_1.docx]

**Supplementary Table 1. Women’s drivers of and trajectories through homelessness**

| **Code** | **Age during firs-time homelessness** | **Predisposing, triggering, perpetuating factors and patterns of pathways between stages** | **Trajectories** |
| --- | --- | --- | --- |
| P1 | 11 | Death of mother due to cancer **→** Group living with peers in rental rooms → Ended up on streets → Rental hotels for a night → Street life in makeshift shelter → Rehabilitation centre for substance addiction treatment → Again on the streets → Relapse → Continued street life (16 years of homelessness) | Chaotic route |
| P2 | 27 | Abandoned by her husband while she was 8 months pregnant → Unable to pay a house rent → Stayed with her friend (married friend) → Couple’s conflict → Ended up in the streets → Three months on the streets | Circuitous route |
| P3 | 7 | Parental alcohol abuse and family conflict → Left home and ended up on the streets → Rapped and impregnated → Son stolen → Started a relationship and lived in a rental room → Gave birth again − left by a partner → Returned to street life → 18 years of homelessness | Chaotic route |
| P4 | 26 | Attempted abduction before the age of 9 → Migrated to Addis Ababa to escape abduction → Worked as a housemaid for a year → Group living in a rented room → started life with a partner → Began living together with a man → Conflict → Left by a partner → Unable to pay for the rent → ended up in the streets with two children → Rapped by a complete stranger → 1 year on the street (4 months pregnant at the time of the data collection) | Circuitous route |
| P5 | 18 | Abducted before her menarche → Her husband drinks too much areke → Domestic violence → ended up on the street → Twelve years in the streets 12 years | Linear route |
| P6 | 25 | Family conflict→ left home→ Substance abuse→ Difficulty paying bills → Ended up on the streets→ 2 years | Linear route |
| P7 | 19 | Neglected by parents→ Ran away from home to Addis Ababa → Ended up on the streets→ Raped by two men → Worked for free as a maid → Abuse and food withholding → Escaped from the employer→ Started living in the streets again → Repeated rape and unwanted pregnancies→ 20 years on the streets | Chaotic route |
| P8 | 15 | Raped at the age of 15 → Became pregnant → Moved to Addis Ababa → Fear of disclosure about rape → Started to live in the streets since the age of 15 years → On the streets for 10 years now | Circuitous route |
| P9 | 22 | Worked as a domestic worker since childhood→ Got married→ Husband left her and disappeared → Went out into the street and has been on the street for 3 years now | Circuitous route |
| P10 | 25 | Mother and father separated → Started working as a housemaid → Suffered maltreatment by her employer→ Forced to work for free → Changed homes as a housemaid but was physically abused→ Left home and was brought to Addis Ababa from Arbaminch → Worked as a housemaid for three years → Started living with her partner → Got pregnant → Left by her partner at 6 months of pregnancy → Unable to pay the rent and feed herself → Ended up on the streets for 5 months | Circuitous route |
| P11 | 25 | Family poverty → Housemaid (for fifteen or thirty birr)→ Got married at 20 → Gave birth to 3 children→ Death of her spouse→ Unable to pay for the house rent→ Ended up on the street with children for 5 years | Circuitous route |
| P12 | 20 | Parental divorce → Started living with her mother→ Raped and became pregnant at the age of 16 or 17 years by a neighbour → Family remined silent about the rapist and no measure was taken → Raised her child at family’s home → Family conflict → Left family home and moved to Addis Ababa → Worked as a domestic worker → Went back home → Started working as a domestic worker again→ In a relationship with another man→ He left her when he learnt that she was pregnant → Continued working as a maid→ Conflict with employer→ Started another relationship → Left by the second man when she was 6 months pregnant → Ended up on the streets→ For 6 years | Chaotic route |
| P13 | 12 | Left home by her choice (she is from a wealthy family) → Worked as a domestic worker since the age of 12 → Severe physical abuse by her female employer → Escaped from abuse and started living on the streets → Started smoking, chewing khat, and drinking liquor immediately → Gave birth at the age of 14 (on the streets) → Left by a partner (a street-dweller) → Her daughter raped at the age of 14 by a stranger → Has lived on the streets for 15 years | Chaotic route |
| P14 | 11/12 | Loss of father before the age of 11→ Physically abused by her brother → Maternal favouritism continued → Left family home at the age of 11 or 12 → Attempted rape→ Raped at the age of 14 → Justice not served→ Suicidal attempt after the rape → 14-year-old onset of substance use → Being in a relationship with a street dweller→ Gave birth at the age of 16 → Experienced abuse by a partner→ Continuing substance use and assault → Escaping from him → Was with another man→ Her children experienced verbal violence by their stepfather → Left the new partner → Had been living on the streets for 16 years now | Chaotic route |
| P15 | 20 | Loss of her mother before the age of 7→ Working as a domestic worker at the age of 7→ Unable to bear household chores above her capacity → Brought by her sister to Addis Ababa→ A domestic worker again→ Struggling with high workload and dropped out from night school→ Started to live with a partner → Left by her partner during pregnancy → Ended up in the streets→ 5 months | Circuitous route |
| P16 | 20 | Abducted and married at fifteen → Too much pressure from her husband’s family → Selling wood and raising children → Partner’s alcohol abuse→ Domestic abuse → Went out on the street → Left by her partner during pregnancy→ Had been living on the street for 6 years | Circuitous route |
| P17 | 18 | Death of her father at an early age → Mother married another person → Mother left her daughter with her uncle’s wife → The mother hid her address from her daughter → Forced to do domestic work by her aunt → Beaten when too tired to work → Food withheld → Left home → Found and taken by a stranger woman → Worked as a housemaid without payment → Physical abuse → Left home for the second time → Found her mother's address → Went to her mother’s place → Verbally and physically abused by her stepfather → Glaring and frowning faces → Withholding food→ Mother was not there to protect → Began to live with her uncle→ Got raped and impregnated by him→ Dared not to speak to anyone → Home delivery→ Forced to give her newborn to a police station by her uncle→ Keep the newborn at the organisation for seven months → Begin to work→ Life became hard → Ended up in street life → 5 years | Chaotic route |
| P18 | 29 | Death of her partner→ Life got tough ⎯ moved to Addis Ababa → Engaged in a relationship→ Got pregnant, but the partner left after four months→ Had trouble paying the rent → Lack of social support and ended up begging on the streets → Had been on the street since 1 year | Circuitous route |
| P19 | 12 or 13 | Loss of her father → Mother got married → Stepfather's alcohol abuse→ Physical assault and name-calling→ Leaving school and escaping from home → Moving to her grandfather’s house→ Child labour and physical abuse continued→ Escaped from home→ Brought to Addis Ababa by a nun→ Living with the nun in a cemetery → Street selling and begging → Raped at the age of 13 → Death of the nun → Street life began → First child at 16 years of age → 17/18 years → Onset of substance abuse → Raped by her partner’s friend and became pregnant→ Abortion→ Death of her partner → Depression and substance continued→ 16 years | Chaotic route |
